# Editorial: Cytomegalovirus Pathogenesis and Host Interactions

**DOI:** 10.3389/fcimb.2021.711551

**Published:** 2021-07-07

**Authors:** Emma L. Poole, Michael M. Nevels

**Affiliations:** ^1^ Division of Infectious Diseases, Department of Medicine, University of Cambridge, Cambridge, United Kingdom; ^2^ Biomedical Sciences Research Complex, School of Biology, University of St Andrews, St Andrews, United Kingdom

**Keywords:** human cytomegalovirus, murine cytomegalovirus, innate immune response, adaptive immune response, latent infection, reactivation from latency, productive infection, anti-viral therapy

## Scope of the Research Topic

Human cytomegalovirus (HCMV) is a very widespread and highly prevalent β-herpesvirus, which sometimes causes mononucleosis following primary infection but is rarely associated with severe disease in immunocompetent individuals (Boeckh and Geballe, 2011; Griffiths et al., 2015). However, like all herpesviruses, HCMV establishes infections that last for the life of the host in part by residing in a dormant state referred to as ‘latency’ (Sinclair and Poole, 2014; Dupont and Reeves, 2016). Reactivation from latency or primary infection can cause debilitating damage in unborn children or life-threatening disease in immunosuppressed patients including recipients of solid organ or hematopoietic cell transplants (Collins-McMillen et al., 2018; Heald-Sargent et al., 2020). Besides human cell culture systems of HCMV productive and latent infection (Peppenelli et al., 2021; Poole et al., 2021), mice infected with murine cytomegalovirus (MCMV) have served as invaluable models to understand host immune responses, viral immune evasion strategies and the mechanisms of pathogenesis (Brizic et al., 2018; Reddehase and Lemmermann, 2018). HCMV replicates productively in a wide variety of terminally differentiated cell types (‘lytic’ infection) while targeting select undifferentiated cells, including myeloid progenitors and monocytes, for latent infection (Sinclair and Poole, 2014; Goodrum, 2016). CMVs are highly sophisticated pathogens encoding hundreds of proteins and non-coding RNAs that engage in a myriad of host interactions (Stern-Ginossar et al., 2012; Weekes et al., 2014). The study of these interactions is constantly revealing new and surprising insights into both the replication and persistence strategies of the virus as well as the biology of the host cell and organism.

The recently published Research Topic ‘Cytomegalovirus Pathogenesis and Host Interactions’ combines 28 articles (8 Original Research Articles, 10 Brief Research Reports, 8 Reviews, 1 Mini Review and this Editorial), involving 138 authors, 45 reviewers and 5 editors working in the field. Below, we are providing an overview of the articles published in this Research Topic, dividing them into the four sections ‘innate and adaptive immune control of CMV pathogenesis’ (11 articles), ‘host interactions during CMV latency and reactivation’ (6 articles), ‘host interactions during productive CMV infection’ (6 articles) and ‘targeting CMV pathogenesis by anti-viral therapy’ (4 articles).

## Innate and Adaptive Immune Control of CMV Pathogenesis

The lifelong relationship with HCMV is based on our intrinsic, innate and adaptive immune responses as well as manifold viral countermeasures which collectively enable a dynamic balance between host and pathogen that largely precludes disease without eliminating the virus from our bodies (Berry et al., 2020; Schilling et al., 2021). Major disease or even death caused by HCMV is often linked to inflammation, including pro-inflammatory cytokine production and is usually limited to situations where the immune system is significantly suppressed or still immature (Boeckh and Geballe, 2011; Griffiths et al., 2015).

Viral infections often induce autophagy, which has been considered an intrinsic cellular defense mechanism, and herpesviruses have developed strategies to evade and manipulate this host response (Lussignol and Esclatine, 2017; Tognarelli et al., 2021). López Giuliani et al. report that HCMV inhibits autophagy in renal tubular epithelial cells and promotes cellular enlargement. Their findings have potential implications for HCMV-related kidney disease. As in other infectious pathogens, the innate immune responses to CMV are thought to be triggered by pathogen-associated molecular patterns that engage pattern recognition receptors including Toll-like receptors (TLRs), resulting in the release of anti-viral and pro-inflammatory cytokines (West et al., 2012; Zheng et al., 2020). Frascaroli et al. analyzed TLR genotypes and responses in immunocompetent patients with primary symptomatic HCMV infection presenting as mononucleosis. Although the study identified no difference in TLR2, 3, 4, 7 and 9 single nucleotide polymorphisms between symptomatic and asymptomatic individuals, TLR2 and TLR7/8 responses were altered in patients with CMV mononucleosis compared to healthy control subjects. The former exhibited higher levels of the pro-inflammatory cytokines interleukin 6 (IL-6) and tumor necrosis factor alpha (TNF-α), but not interleukin 10 (IL-10). Interestingly, HCMV encodes its own homologs of cellular IL-10, which is a largely anti-inflammatory and immunosuppressive cytokine (Avdic et al., 2014; Poole and Sinclair, 2015). Poole et al. review the expression, structure and function of the different viral IL-10 isoforms identified to date as well as the effects HCMV confers on the expression of cellular IL-10. Furthermore, Lau et al. identified a novel viral suppressor of pro-inflammatory cytokine production during HCMV infection. They demonstrate that the HCMV long non-coding RNA1.2 antagonizes the expression and release of IL-6 through a mechanism involving the tumor protein p63-regulated gene 1-like protein (TPRG1L) and nuclear factor kappa B (NFκB) but not TNF-α.

Mast cells are part of the innate immune system, acting as first-line sentinels for environmental antigens, but also provide a link to the adaptive immune system by secreting chemokines that recruit CD8+ T-cells to sites of infection (Podlech et al., 2015; Dahlin et al., 2021). Schmiedeke et al. reveal an unanticipated function of the viral mitochondria-localized inhibitor of apoptosis (vMIA) m38.5, encoded by MCMV, in inducing mast cell degranulation.

It has long been known that CD8+ T-cells play a critical role in controlling CMV infection and disease (Klenerman and Oxenius, 2016; Jackson et al., 2019). CMV has evolved a myriad of countermeasures to the CD8+ T-cell response including major histocompatibility (MHC) class I antagonism (Manandhar et al., 2019; Berry et al., 2020). Gabor et al. demonstrate that downregulation of MHC class I molecules by HCMV, although not always complete, occurs during all phases of viral replication. Holtappels et al. present an impressive example of lethal organ disease, due to insufficient antigen presentation resulting from viral immune evasion, after allogenic hematopoietic cell transplantation in MHC class I-mismatched mice infected with MCMV. Along the same lines, deletion of viral immune evasion genes enhanced antigen presentation preventing lethal CMV disease in minor histocompatibility antigen-mismatched hematopoietic cell transplantation (Gezinir et al.). Conversely, Becker et al. confirm that MCMV m04/gp34, originally thought to be an immune evasion protein, is a positive regulator of MHC class I presentation and protection by CD8+ T-cells, reminding us that interactions of CMV with the immune system are rarely unidirectional and sometimes counterintuitive. While the role of HCMV-specific CD8+ T-cells has been extensively investigated, the CD4+ T-cell response received less attention. Lim et al. review the significant recent advances in our understanding regarding the importance and contribution of CD4+ T cells to anti-viral immunity and life-long carriage in both healthy and immunocompromised individuals. The same team also presents highly improved functional assays that directly assess the cell-mediated immune responses to HCMV in transplant recipients and healthy controls (Houldcroft et al.).

## Host Interactions During CMV Latency and Reactivation

Latency is defined as the maintenance of viral genomes in the reversible absence of virus particle production. That said, how CMV genomes are maintained during latency is unknown (Goodrum, 2016; Adamson and Nevels, 2020). Likewise, we are just beginning to understand the viral and cellular mechanisms that determine the establishment and maintenance of latent CMV infection (Poole and Sinclair, 2020; Shnayder et al., 2020) as well as reactivation from latency (Collins-McMillen et al., 2018; Heald-Sargent et al., 2020).

Mauch-Mücke et al. provide initial evidence for the idea that HCMV episomes are tethered to mitotic host chromosomes in both productively infected fibroblasts and myeloid cells supporting latency. Their data also suggest that viral major immediate-early (IE) proteins of the IE1 family contribute to HCMV episome tethering. These findings are consistent with two other recent reports implicating IE1 family proteins in viral genome maintenance (Tarrant-Elorza et al., 2014; Lyon et al., 2020). A role for IE1 in maintaining HCMV latency may come as a surprise, since the major IE promoter-enhancer (MIEP) appears to be highly repressed during latency in myeloid cells including monocytes (Elder and Sinclair, 2019; Dooley and O’Connor, 2020). In fact, the article by Min et al. recaps the evidence for the view that differentiation of HCMV-infected monocytes is required for major IE gene expression and viral reactivation. However, others have proposed that establishment of latency in myeloid cells is preceded by an initial burst of ‘lytic’ gene expression (O’Connor and Murphy, 2012; Forte et al., 2018). This latter view is shared by Forte et al., who also review recent studies suggesting that the viral genome is more actively expressed during latency than had been anticipated (Cheng et al., 2017; Shnayder et al., 2018). This review further reminds us that CMV latency and reactivation are intricately linked with the host immune response. Collins-McMillen et al. review the mechanisms by which recently identified alternative promoters driving expression of IE1 and IE2 may allow the virus to repress viral gene expression for latency while retaining the ability to respond to cell type-specific host cues for reactivation (Arend et al., 2016; Collins-McMillen et al., 2019). pUS28 is another viral protein linked to the maintenance of HCMV latency (Krishna et al., 2017; Krishna et al., 2019). Krishna et al. confirm the role of this protein in viral latency by ruling out that inadvertent effects on the adjacent genes US27 and US29 contribute to the phenotype of US28-deficient viruses. pUS28 is a ‘Swiss army knife’ involved in many processes including calcium regulation (Miller et al., 2012; Krishna et al., 2018). Dunn and Munger review how manipulation of the calcium and 5’-adenosine monophosphate-activated protein kinase (AMPK) signaling network by several HCMV proteins including pUS28 contributes to infection and pathogenesis.

## Host Interactions During Productive CMV Infection

Reactivation from latency or primary infection is concomitant with the CMV productive cycle that proceeds through successive IE, early and late stages resulting in the production of infectious progeny. In the pre-IE stage, proteins and RNAs packaged within the virion tegument are delivered to the host cells to prime them for productive infection (Kalejta, 2008; Penkert and Kalejta, 2011).

Kalejta and Albright review both established and emerging functions of pp71 (pUL82), one of the most prominent tegument proteins of HCMV, and their implications for pathogenesis and vaccine development. A key function assigned to pp71 is activation of the MIEP, which drives IE1 and IE2 expression (Torres and Tang, 2014; Landolfo et al., 2016). Previous work has shown that the essential 86-kDa IE2 protein of HCMV accelerates viral gene transcription while maintaining the steady-state levels below a cytotoxic threshold (Teng et al., 2012; Vardi et al., 2018). Chaturvedi et al. report that this ‘accelerator’ circuit is shared between IE2 and infected cell protein 4 (ICP4) of Herpes simplex virus type 1, suggesting an evolutionary conserved strategy. Another essential DNA binding regulator of viral gene expression is the HCMV pUL34 protein (Rana and Biegalke, 2014; Slayton et al., 2018), and MCMV encodes the homologous protein M34. Eilbrecht et al. demonstrate that M34 is expressed with early-late kinetics, localizes to the host cell nucleus and promotes viral replication, albeit in a non-essential way.

Besides focused analyses of individual viral gene products, systems approaches are being increasingly deployed for a broader view of how the hundreds of CMV proteins and non-coding RNAs interact with the host cell (Landais and Nelson, 2013; Poole and Sinclair, 2020). Lee and Grey review how high-throughput screening approaches have evolved and resulted in a new understanding of the virus-host interactions during productive HCMV infection. These high-throughput approaches include proteomics studies revealing degradation of far more than a hundred host proteins shortly after infection (Weekes et al., 2014; Nobre et al., 2019). For their contribution, Lin et al. employed an inhibitor-based proteomics screen to find that the predominant mechanism of host protein degradation by HCMV is *via* the proteasome, although alternative degradation pathways are also relevant. One of these degraded host proteins appears to be the sterile alpha motif and histidine-aspartate domain containing protein 1 (SAMHD1), which has been recently identified as a restriction factor of both MCMV and HCMV (Businger et al., 2019; Kim et al., 2019). The article by Hyeon et al. confirms that SAMHD1 inhibits HCMV replication and concludes that degradation of this host protein in the late phase of infection requires Cullin-RING-E3 ligase complexes.

## Targeting CMV Pathogenesis by Anti-Viral Therapy

In the absence of an effective vaccine, the prevention or treatment of HCMV disease largely relies on a few small molecule compounds with anti-viral activity. However, current antivirals for HCMV come with major downsides that include poor bioavailability, adverse side effects and the emergence of resistant virus strains, calling for new molecular targets and treatment strategies (Adamson and Nevels, 2020; Bogner et al., 2021).

Suárez et al. applied high-throughput sequencing coupled with HCMV-adapted target enrichment to samples collected from recipients of solid organ and hematopoietic cell transplants undergoing anti-viral therapy. Six different resistance mutations in the viral DNA polymerase (UL54) gene were detected in six out of eleven patients in this study. These results highlight the potential use of high-throughput sequencing to monitor strain composition and resistance to antivirals.

A promising new area of translational research and drug development is ‘epigenetic therapy’ (Nevels et al., 2011; Nehme et al., 2019). Groves et al. review the anti-viral activity of small molecule inhibitors targeting the bromodomains of bromo- and extra-terminal domain (BET) proteins, which serve as ‘readers’ of acetylated histones. Bromodomain inhibitors show promise as a novel class of therapeutics for disease linked to infection with HCMV and other human herpesviruses. The identification and characterization of new anti-viral drug candidates require sensitive and reliable detection techniques of HCMV infection, gene expression and spread. Rand et al. present a multi-reporter HCMV, identified through iterative testing of minimally invasive mutations, expressing three distinct fluorescent reporter genes that permit the visualization of infected cells in the IE, early and late phases of infection. They also validate their approach in identifying anti-viral compounds and narrowing down their mechanism of action in a single step.

An alternative treatment approach for HCMV disease, besides anti-viral drugs, is the adoptive transfer of CD8+ T-cells (Mui et al., 2010; Garcia-Rios et al., 2021). Renzaho et al. demonstrate that CD8+ T-cells not only prevent lethal MCMV disease by limiting viral spread in vital organs, but also control infection of stromal cells in the bone marrow. Thus, CD8+ T-cell transfer allowed successful donor hematopoietic stem cell engraftment, confirming cyto-immunotherapy as a viable option to target CMV infection after hematopoietic stem cell transplantation.

## Concluding Remarks

This article collection reflects some of the astounding breadth, current focus areas and future directions in HCMV and MCMV research. We thank all the authors, reviewers and guest editors who contributed to this project, which, in part due to the ongoing pandemic, took more than a year to complete. The Research Topic has received 71,531 total views as of 1st July 2021, and we hope that the included articles will receive much attention and will inspire plenty of further CMV research.

## Author Contributions

MN and EP wrote the manuscript and approved the submitted version.

## Funding

MN and EP are funded by the Medical Research Council (MR/P022146/1 and MR/S00081X/1, respectively).

## Conflict of Interest

The authors declare that the research was conducted in the absence of any commercial or financial relationships that could be construed as a potential conflict of interest.
